# Multibody Models for the Analysis of a Fall From Height: Accident, Suicide, or Murder?

**DOI:** 10.3389/fbioe.2019.00419

**Published:** 2019-12-12

**Authors:** Giulia Pascoletti, Daniele Catelani, Paolo Conti, Filippo Cianetti, Elisabetta M. Zanetti

**Affiliations:** ^1^Department of Engineering, University of Perugia, Perugia, Italy; ^2^MSC Software Corporation, Turin, Italy

**Keywords:** fall, accident, forensic, crime, android, multibody, doe, biomechanics

## Abstract

The final subject position is often the only evidence in the case of the fall of a human being from a given height. Foreseeing the body trajectory and the respective driving force may not be trivial due to the possibility of rotations and to an unknown initial position and momentum of the subject. This article illustrates how multibody models can be used for this aim, with specific reference to an actual case, where a worker fell into a stair well, prior to stair mounting, and he was found in an unexpected posture. The aim of the analysis was establishing if this worker was dead in that same place, if he had been pushed, and which was his initial position. A multibody model of the subject has been built (“numerical android”), given his stature and his known mass. Multiple simulations have been performed, following a design of experiments where various initial positions and velocity as well as pushing forces have been considered, while the objective function to be minimized was the deviation of the numerical android position from the actual worker position. At the end of the analysis, it was possible to point how a very limited set of conditions, all including the application of an external pushing force (or initial speed), could produce the given final posture with an error on the distance function equal to 0.39 m. The full analysis gives a demonstration of the potentiality of multibody models as a tool for the analysis of falls in forensic inquiries.

## Introduction

Fall from height has a significant incidence among work-related injuries, reaching over 40% for the construction industry (Dong et al., 2017). The reconstruction of the accident might be fundamental whenever the initial position of the worker needs to be established in order to assess his own responsibility, or in those cases where the suspect of murder or suicide does exist. Legal medicine can give a substantial support in these inquiries, assessing the injuries severity, and the consequent required input energy (Atanasijevic et al., 2015; Rowbotham and Blau, 2016; Rowbotham et al., 2018). On the other side, biomechanical analyses can provide more detailed information about body kinematics (and even body segments kinematics) through the use of suitable experimental or numerical models (Muggenthaler et al., 2013). The initial worker position and velocity being unknown, these sort of analyses are performed through multifactorial design of experiments where multiple sets of initial conditions are tested as far as the final outcome complies with the empirical evidence, which more often is the only final body position or eventual testimonies. Experimental models are based on the use of anthropomorphic test devices (Cao et al., 2016) and are affected by some major shortcomings: existing validated dummies are expensive; they represents an “average” anthropometry since they cannot reproduce the actual anatomy of the victim therefore some sort of generalization is required; they provide a limited set of information [from sensors or markers acquired through multiple cameras (Seacrist et al., 2010)]; experiments are time-consuming. On the other side, numerical models overcome most of the above cited limitations, but they need to be validated in order to produce reliable results; as such, a combined approach where experimental data from anthropomorphic dummies are used to set up numerical models is the most promising (Büchner et al., 2019).

Numerical models to be used for dynamic analyses are made of masses, connected to one another through joints, simulating skeletal articulations. These joints may have linear or, more often, non-linear elastic behaviors which are able to produce more accurate results (Richard et al., 2016). Another recent advancement in these models is including some deformable bodies (Terzini et al., 2017; Zanetti et al., 2017, 2018; Pascoletti et al., 2018; Putame et al., 2019) for those bodies which are likely to undergo relevant deformations.

Multibody models have one more advantage over anthropomorphic dummies that is the possibility of simulating voluntary movements produced by muscle activation [(Milanowicz and Kedzior, 2017) “active models”]. This possibility should be exploited with caution since it is impossible to foresee which voluntary reactions could a human being have in the short time of an accident: extensive experimental tests are needed in order to produce reliable muscle activation patterns, corresponding to unconditional reflexes (Devane et al., 2019). A sensible way of proceeding may be using passive models for a first screening, and implementing active models only if the first ones have proved to be inadequate.

The case here analyzed refers to the fatal fall from height of a man at work. A clinical trial followed and the judge appointed one of the authors as a prosecutor to establish if it was possible for the victim to fall and land where the cadaver was found, or if the cadaver had probably been moved from elsewhere else. Secondly, the prosecutor had to establish if such a fall needed a voluntary action (murder or suicide) or it could be simply due to a fatal accident; the authors have tried to give an answer to these questions through a numerical analysis, based on a multibody model. The model has been described in detail, especially with reference to the simulation of articular joints since joint stiffness and contact parameters have been seldom reported in a systematic way, while their knowledge is mandatory in order to be able to discuss the respective model behavior compared to other works in literature. The numerical analysis has produced a new insight into the accident kinematics, providing valuable information for the forensic dispute. The model introduced can be generalized to study different body anthropometries thanks to regression functions, allowing to calculate mass and geometries from subject weight and height (Robbins, 1983). In addition, different environments can be easily simulated.

## Materials and Methods

The authors have chosen to use a numerical multibody model (MSC Adams software v. 17, by MSC Software Corporation): the subject body is made of rigid segments, with mass and inertial moments assigned to each of them; all segments are articulated to one another through elastic joints. The initial conditions of each part belonging to the android articulated model (in terms of the respective position and speed of the center of mass) have been established through a design of experiments (DOE); the known outcome on which this analysis was based had to be the final position of the body, according to pictures and measurements taken by legal prosecutors.

In the following the multibody model is described in details, as well as input variables for DOE and their respective range of variation. Finally, the objective function, used to measure the “goodness of fit” of the supposed fall kinematics, is reported.

### Description of the Numerical Model

**Figure 1:**
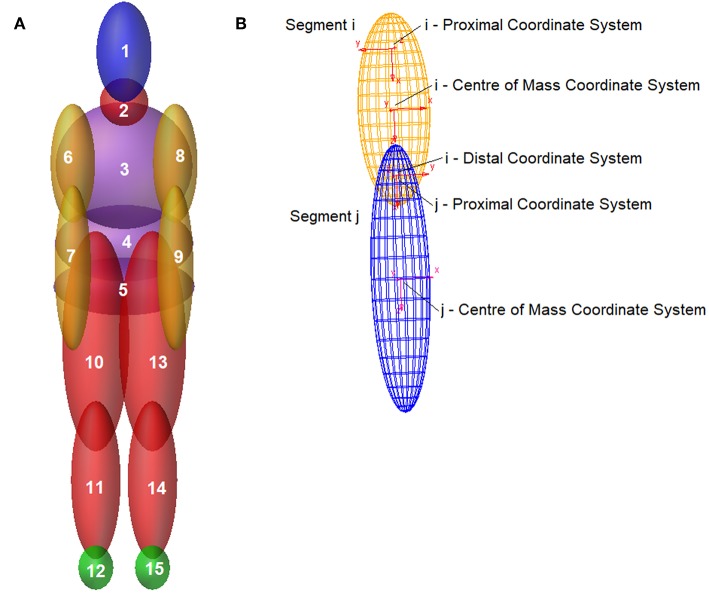
**(A)** Android Model's Segments—**(B)** Segments reference coordinate systems.

The articulated total body model is made of 15 ellipsoidal elements, connected to one another by means of 14 joints, as detailed in Figure 1A and Table 1.

**Table 1:** Segments description.

**Segment number**	**Segment name**
1	Head
2	Neck
3	Upper Torso
4	Central Torso
5	Lower Torso
6	Right Upper Arm
7	Right Lower Arm
8	Left Upper Arm
9	Left Lower Arm
10	Right Upper Leg
11	Right Lower Leg
12	Right Foot
13	Left Upper Leg
14	Left Lower Leg
15	Left Foot

Ellipsoids geometry is completely defined by a center of mass coordinate system and two coordinate systems located, respectively, at the proximal and distal ends. The respective geometry is detailed in Figure 1B, where the joint between two adjacent segments is shown. The location and orientation of connection joints are defined by the *i* distal and *j* proximal coordinates systems (Figure 1B).

Body segments have been assigned also a mass and inertial properties, according to anthropomorphic measurements referred to the fiftieth percentile having the input weight and height [UMTRI reports (Robbins, 1983).

Simple mechanical joints or more complex joints (generated as a combination of simple ones) have been used to reproduce natural human joints with the respective degrees of freedom (DOF). More in detail, three type of constrains have been applied: spherical (DOF: 3 rotations), revolute (DOF: 1 rotation), and primitive perpendicular (DOF: 2 rotations), as described in Table 2.

**Table 2:** Mechanical—Body joints correspondence.

**Mechanical joint**	**DOF**	**Body joint**
Spherical	3 Rotations	Upper Neck
Spherical	3 Rotations	Lower Neck
Spherical with Perpendicular	2 Rotations (rotation along the long axis segment is removed)	Right/Left Shoulder
Revolute	1 Rotation in the sagittal plane	Right/Left Elbow
Spherical	3 Rotations	Lumbar Spine
Spherical	3 Rotations	Thoracic Spine
Spherical with Perpendicular	2 Rotations (rotation along the long axis segment is removed)	Right/Left Hip
Revolute	1 Rotation in the sagittal plane	Right/Left Knee
Revolute	1 Rotation in the sagittal plane	Right/Left Ankle

Axial rotations around the long bone's axis and abduction/adduction movements of elbows, knees and ankles have not been taken into consideration in the following simulations, in order to simplify the model, in relation of its purpose. Indeed, preliminary tests have demonstrated that these movements did not take place or were very limited for this case study.

The passive resistance of all joints has been defined. This job has represented a major burden in the modeling process, due to the high number of degree of freedoms involved, and to many different analytical laws having been implemented in literature, sometimes with peculiar reference systems. After a wide literature survey (Engin, 1979; Engin and Chen, 1987; Bergmark, 1989; Riener and Edrich, 1999; Haug et al., 2004; Sharan et al., 2013), a non-linear formulation of moment/rotation law has been here chosen, with few exceptions. Joint passive resistance must accomplish a double role:

- Limiting the joint range of motion
- Joint stabilization, preventing segments collapsing under their own weight.

Table 3 reports in detail, joint by joint, the passive stiffness behavior for all DOFs as well as the respective range of motion. All resistive parameters (curves and ranges) have been obtained experimentally in literature (as detailed in the first column) and values were derived from interpolation or regression of experimental data. Range of motion (ROM) values here reported represent the maximum angular limits which can be reached when a quasi-static rotation is applied, according to cited literature. Angle ranges are actually smaller than those measured for passive activation: it is well-known, for example, than muscle activation takes place during a fall in order to prepare for “landing” (Santello et al., 2001; Pontaga, 2004; Strimpakos, 2011); for this reason smaller ranges have been implemented in the models for selected joints, according to validation experiments described in sections “Validation of the numerical model” and “Model validation results.”

**Table 3:** Passive resistive moments characteristics.

**Human joint**	**Joint movement**	**Range of motion (ROM)**	**Resistive moment [Nm]**	**Stiffness value [Nm/^**°**^]**	**Damping coefficient [Ns/m]**
Upper/Lower Neck (Haug et al., 2004)	Flexion	0°-30°		1.4	0.0678
	Extension	0°-35°		2.5	
	Lateral Bending	0°-45°		2.2	
	Twist	0°-50°		0.5	
Shoulder (Engin, 1979)	Flexion/Extension	−50°−180°	$e^{\left(3.3671*\left(-\theta_{S}-0.2543\right)\right)}+-e^{\left(-3.5743*\left(-2.1966+\theta_{S}\right)\right)}$		0.0678
	Abduction/Adduction	−50°-160°	$0.77-9.21\theta_{s}+4.99{\theta_{s}}^{2}+5.46{\theta_{s}}^{3}++0.86{\theta_{s}}^{4}-10.12{\theta_{s}}^{5}+6.42{\theta_{s}}^{6}+-1.18{\theta_{s}}^{7}$		
	Abduction in Frontal Plane	0°-160°	$-592.67+1766.31\theta_{s}-2070.46{\theta_{s}}^{2}++1190.19{\theta_{s}}^{3}-335.65{\theta_{s}}^{4}+37.28{\theta_{s}}^{5}$		
Thoracic (Bergmark, 1989; Sharan et al., 2013)	Flexion	0°-10°		3	0.0565
	Extension	0°-5°		3.4	
	Lateral Bending	0°-20°		2	
	Twist	0°-30°		2.5	
Lumbar (Kapandji, 2007; Sharan et al., 2013)	Flexion	0°-45°		1.8	0.0565
	Extension	0°-10°		2.5	
	Lateral Bending	0°-20°		1.3	
	Twist	0°-5°		0.9	
Elbow (Engin and Chen, 1987)	Flexion	0°-150°	$e^{\left(8.7084*\left(-\theta_{E}+0.1201\right)\right)}+{-e}^{\left(9.4336*\left(-2.3187+\theta_{E}\right)\right)}$		0.0339
Hip (Riener and Edrich, 1999; Haug et al., 2004)	Flexion/Extension	−30°-150° [−30°−50°]	$e^{\left(1.4655-\left(0.0034\theta_{K}-0.075\theta_{H}\right)\right)}+-e^{\left(1.3403-0.0226\theta_{K}+0.0305\theta_{H}\right)}+8.072$		0.0339
	Abduction in the Frontal Plane	0°−80°		1.2	
	Adduction in the Frontal Plane	0°−30°		0.8	
Knee (Riener and Edrich, 1999)	Flexion	0°-150°	$e^{\left(1.8-0.0460*\theta_{A}-0.0352*\theta_{K}+0.0217*\theta_{H}\right)}+-\;e^{\left(-3.971-0.0004*\theta_{A}+0.0495*\theta_{K}-0.0128*\theta_{H}\right)}+-4.820+e^{\left(2.220-0.150*\theta_{K}\right)}$		0.0339
Ankle (Haug et al., 2004)	Plantar flexion	0°-50°		0.3	0.0339
	Dorsiflexion	0°-30°		0.5	

*θ_S_: shoulder flexion/extension angle*.

*θ_s_: shoulder abduction angle*.

*θ_E_: elbow flexion angle*.

*θ_H_: hip flexion/extension angle*.

*θ_K_: knee flexion/extension (in the application of this formula for the flexion/extension resistance of the hip, this angle has been set equal to zero)*.

**Figure 2:**
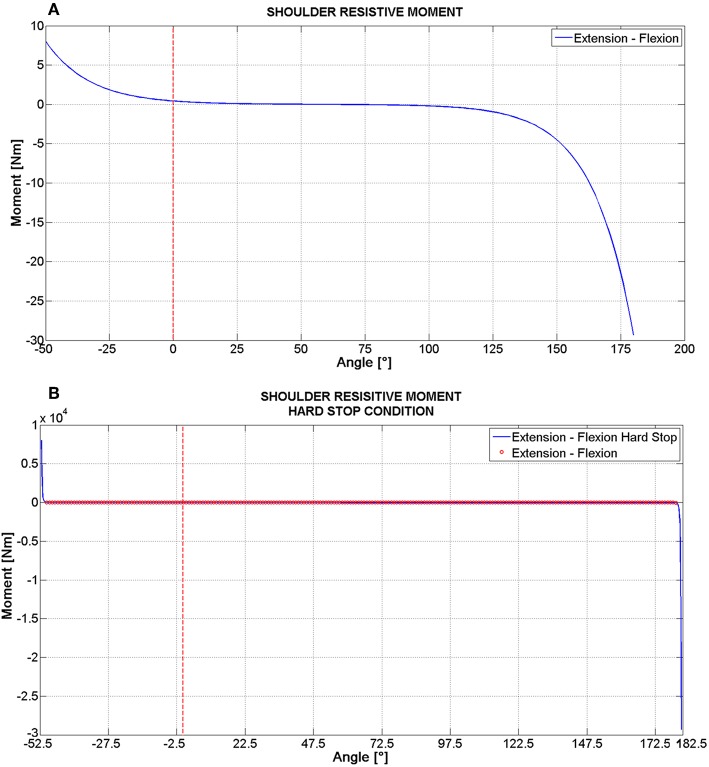
Passive resistive moment for shoulder flexion/extension: the general trend including a “Hard stop” **(B)** and a zoomed view **(A)**.

Whenever the passive joint resistance has been modeled with a non-linear behavior, the force/displacement function was analytically described through a spline, whose trend is similar to the one reported in Figure 2, referring to shoulder flexion/extension. Given the null rotation condition (which has been defined with reference to the straight standing position for all joints, Figure 1A), there is a range of angles, within the joint's ROM, where the resistive torque is very low (near to zero). At the end values, that is when the joint's rotation is close to the extreme of the ROM, the resistive torque increases sharply. In addition to this behavior, which is similar to those reported in literature (Engin, 1979; Riener and Edrich, 1999; Prasad et al., 2010), the numerical simulation has required adding a “hard stop” condition (Figure 2B) in order to effectively limit the range of motion of each joint, without adding angular constrains. According to this condition, when an extreme angle of rotation is approached, the torque value increases up to 1,000 times its value, within a motion of 2°. This is the reason of the very steep spline reported in Figure 2B. The same criterium has been followed when a linear stiffness model has been adopted: whenever the ROM limit is reached, the rotational stiffness rises up to 10,000 Nm/° within 2° rotation.

Joint resistive properties have been completed with constant viscous damping coefficients (Table 3), retrieved from literature (Cheng et al., 1998); they have been introduced to prevent unrealistic vibrations.

### Validation of the Numerical Model

The numerical model has been validated for one specific anthropometry, comparing its results with experimental results obtained by Hajiaghamemar et al. (2015) with a Hybrid III anthropomorphic dummy. In this study five simple scenarios of a fall have been tested and head impact parameters have been calculated. Scenario 1 reproduces a backward fall with no rotation of hip joints before the head hits the ground; scenario 2 is a backward fall with hip flexion, where the head impacts the ground after the hips; scenario 3 represents a forward fall with knees flexion and these hit the ground before the head; scenario 4 reproduces a forward fall with knees fixed and scenario 5 is a sideward fall where shoulder first contacted the ground.

**Figure 3:**
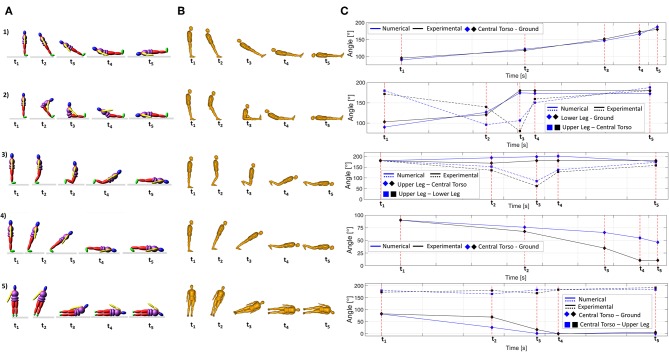
Scenario 1, Scenario 2, Scenario 3, Scenario 4, Scenario 5: **(A)** Numerical simulation—**(B)** Experimental data from Hybrid III dummy (Reprinted by permission from Springer Nature Customer Service Centre GmbH: Springer Nature, Hajiaghamemar et al., 2015)—**(C)** Comparison of angles' variations.

These same configurations have been simulated with the developed model (Figure 3), where segment masses and geometry were chosen from dummy height and weight (Robbins, 1983), and results have been compared (Table 4). With reference to scenario 5, stiffness properties (for shoulder and elbow) and contacts associated to the left arm have been deactivated through the specific function in Adams, in order to reproduce the experimental setup (Hajiaghamemar et al., 2015) and to allow the head to impact the ground.

**Table 4:** Head impact force.

	**Head impact force [kN]**				
	**Scenario 1**	**Scenario 2**	**Scenario 3**	**Scenario 4**	**Scenario 5**
Experimental (Dummy)	22.8 ± 2.1	14.9 ± 4.6	20.3 ± 3.7	21.6 ± 6.1	17.1 ± 2.2
Simulation (Model)	22.9	14.83	21.46	24	18.6
Analytical deviation	Δ = 0.1 [*kN*] Δ% = 0.44%	Δ = −0.07 [*kN*] Δ% = −0.47%	Δ = 1.16 [*kN*] Δ% = 5.7%	Δ = 2.4 [*kN*] Δ% = 11%	Δ = 1.5 [*kN*] Δ% = 8.8%

These five scenarios have been realized applying suitable motion laws to joints for the first few instants, and the only gravity action was simulated from that point on.

### Design of Experiments

**Figure 4:**
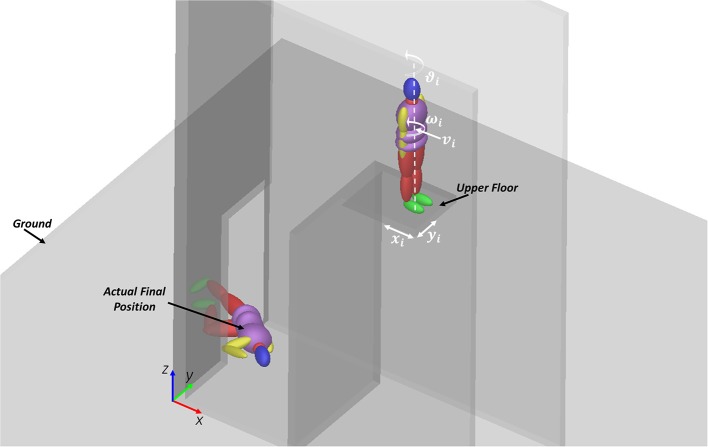
Initial parameters definition and actual scenario representation.

The choice of input parameters to be varied, according to the design of experiments, has not been trivial, since it was necessary to list all unknown variables, and to select a limited set of those variables which were likely to play a significant influence on the final victim position. According to first trials, the authors have chosen to consider five variables, defining the body position on the upper floor, its orientation, and the initial speed of the central torso (which simulates an impulsive action due to a shove); the respective representation is reported in Figure 4; while the range of variation of each input variable is detailed in Table 5. A full factorial plane where each variable could assume three levels has been performed as a first step (243 experiments); according to its results, a new full factorial plane has been designed on a reduced set of variables with five levels each.

**Table 5:** Input variables of DOE.

**Parameter**	**Range of variation**
*x_*i*_ [m]*	0.00 : 1.00
*y_*i*_ [m]*	−0.25 : 0.15
*ϑ_*i*_ [°]*	−90 : 90
*v_*i*_ [m/s]*	−10.00 : −0.10
*ω_*i*_ [°/s]*	−10 : 10

### The Objective Function

The objective function to be minimized was the distance between the actual victim position (“A” configuration in the following) and the position of the multibody android at the end of the simulation (“M” configuration in the following). Seven different functions have been tested in order to choose the best formulation that is the simplest one, leading to the same results as the most complex one. It can be so defined:

$$\begin{array}{l} \text{OBJ}=\overset{\text{n}}{\underset{\text{i}=1}{\sum }}\sqrt{{\left({\text{x}}_{Ai}-{\text{x}}_{\text{Mi}}\right)}^{2}+{\left({\text{y}}_{\text{Ai}}-{\text{y}}_{\text{Mi}}\right)}^{2}} \end{array}$$

Where:

- *x*_*Ai*_, *y*_*Ai*_ are the coordinate of the center of mass of “i” body segment (Figure 1B), with reference to the victim position, as reported by legal prosecutors (Figure 4);
- *x*_*Mi*_, *y*_*Mi*_ are the coordinate of the center of mass of “i” segment belonging to the multibody android model (Figure 1B), with reference to its final position at the end of the simulation;
- *n* is the last body segment being considered.

Values to be assigned to “i” are detailed in Table 6, according to the objective function being considered.

**Table 6:** Body segments considered by each objective function.

		**Body segments**														
		**1**	**2**	**3**	**4**	**5**	**6**	**7**	**8**	**9**	**10**	**11**	**12**	**13**	**14**	**15**
**Objective functions**	OBJ 1	x														
	OBJ 2	x									x			x		
	OBJ 3	x					x		x		x			x		
	OBJ 4	x					x		x		x	x		x	x	
	OBJ 5	x			x						x			x		
	OBJ 6	x			x	x					x			x		
	OBJ 7	x			x	x	x		x		x			x		

## Results and Discussion

### Model Validation Results

The validation of the model has been performed comparing numerical model results with experimental results obtained by Hajiaghamemar et al. (2015) with a Hybrid III dummy. First of all, the model has been validated from the kinematic point of view analyzing the movements of body segments for the five different scenarios for a fall from a standing position, previously described in section “Validation of the numerical model.” This comparison was focused on the analysis of body positions, checking if joints' rotations had been properly limited and that the sequence of segments impact to the ground was the same between the numerical model here developed and the dummy model used in literature (Hajiaghamemar et al., 2015). In Figure 3 results are shown: similarities between numerical and experimental results are stressed both in terms of joints' kinematics and of the sequence of impact, when it is relevant. Figures 3A,B show the sequence of the fall for the numerical and for the experimental model; Figure 3C represents significant angles variations from instant t_1_ to instant t_5_, as extracted from both models. As can be seen, trends of these curves are very similar as well as rotations' values.

The model has proved to be able to simulate both body kinematics and the respective impact forces with a maximum peak error equal to 11% (Table 4). This validation has allowed properly tuning model parameters: for example, with reference to scenario 1, the upper torso joint had to be stiffened otherwise it was the first to impact the ground, reducing the head impact force (which initially resulted to be equal to 15 kN).

### Definition of Input Variables and Selection of the Objective Function

**Figure 5:**
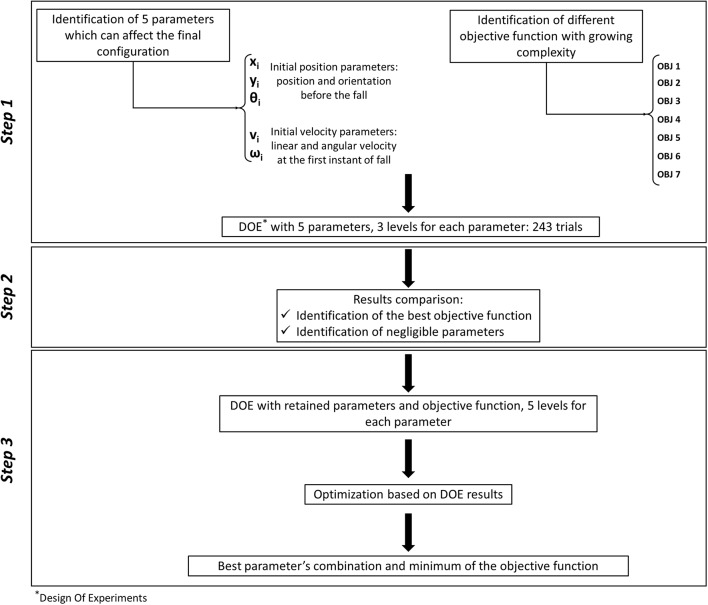
Optimization process workflow.

Figure 5 shows the workflow of the optimization process, whose results will be detailed in the following.

As specified in the above section, the first design of experiments was performed considering five input variables (Table 5), and seven different formulations for the objective function (Table 6); the respective results have been reported in Table 7.

**Table 7:** Preliminary results.

		**Input variables**					**Results**
	***Trial #***	***ϑ_i_*[°]**	***v_***i***_ [m/s]***	*****ω_ι_** [^**°**^/s]***	***x_***i***_ [m]***	***y_***i***_ [m]***	***Obj value [m]***
OBJ1	156	0.0	−0.10	10	0.0	0.15	0.14
OBJ2	137	0.0	−0.10	−10	0.0	−0.05	0.63
OBJ3	137	0.0	−0.10	−10	0.0	−0.05	0.92
OBJ4	137	0.0	−0.10	−10	0.0	−0.05	1.7
OBJ5	127	0.0	−5.05	10	0.0	−0.25	0.80
OBJ6	127	0.0	−5.05	10	0.0	−0.25	0.82
OBJ7	119	0.0	−5.05	0.0	0.0	−0.05	1.33

According to preliminary results, the following statements can be made:

◦ OBJ2, OBJ3, and OBJ4 reach their minimum value for the same set of input parameters (Trial 137); therefore, considering also the center of mass of upper arms or of lower legs is not relevant.
◦ OBJ5 and OBJ6 reach their minimum for the same combinations of parameters. Therefore, the addition of the lower torso center of mass to the objective function is not relevant for the analysis.
◦ All objective functions reach their minimum for ϑ_i_ equal to zero (the android position is on the back, with respect to the aperture).
◦ OBJ1 can reach its minimum value also for incorrect final body positions such as supine or with feet-to-head vector pointing to the door, that is opposite to the x-axis direction (see the reference system in Figure 4).

Taking into account all these observations, the next analysis has been focused on three objective functions, that are OBJ2, OBJ5, and OBJ7. A new analysis has been performed considering these three objective functions; however ϑ_i_ range of variation has been set equal to −5° and 15°, since the previous analysis had demonstrated that its optimized value was zero, and this result was confirmed by optimization analyses which always produced values close to zero. The new design of experiments has produced results reported in Table 8.

**Table 8:** Second DOE results.

		**Input variables**					**Results**
	***Trial #***	***ϑ_***i***_ [^**°**^]***	***v_***i***_ [m/s]***	*****ω_*i*_** [^**°**^/s]***	***x_***i***_ [m]***	***y_***i***_ [m]***	***Obj value [m]***
OBJ2	201	15	−5.05	0.0	0.0	0.15	0.39
OBJ5	201	15	−5.05	0.0	0.0	0.15	0.42
OBJ7	201	15	−5.05	0.0	0.0	0.15	0.71

The results of this second factorial analysis can be so summarized:

◦ OBJ2, OBJ5, and OBJ7 reach their minimum value for the same set of input parameters (Trial 201); therefore, considering the only center of mass of the head and upper legs allows to reach accurate results;
◦ Even when ω_*i*_ varies between its extreme values, the respective objective functions variation is below 2%; as such, input variable ω_*i*_ has been removed from the analysis since it did not play a significant influence (in relation to the hypothesized range of variation).

### Final Results

**Figure 6:**
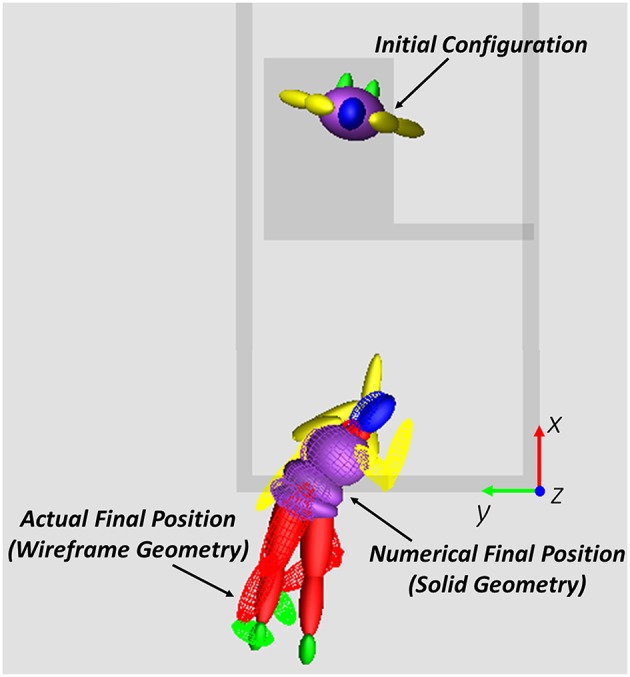
Initial and final configuration for the best parameters' combination: the wireframe model represents the actual victim position, the solid model represents the numerical android position at the end of the simulation.

In the final analysis, the DOE retained four factors and assigned five levels to each of them, for a total number of trials equal to 625, and the objective function OBJ2 was calculated. According to results, the best input variables set is the one reported in Table 9 and the respective result is depicted in Figure 6 (all the falling sequence for the optimum parameters combination is shown in the Video 1 provided in the Supplementary Material section).

**Table 9:** Final results.

**Objective function**	***ϑ_***I***_ [^**°**^]***	***v_***i***_ [m/s]***	***x_***i***_ [m]***	***y_***i***_ [m]***	***Obj value [m]***
OBJ2	15	−5.05	0	0.15	0.39

Even if the final value of the objective function (0.39 m) may not seem so low, it should be reminded that it is a sum of three distances: 0.05 m for the head, 0.15 m for the left upper leg and 0.18 m for the right upper leg.

With the reported “optimal” combination of parameters, the maximum segment distance was obtained for lower arms segments (Figure 6), reaching 0.45 m for the left segment and 0.40 m for the right one.

The computational effort required for all the performed simulations was in general very low (PC with i7-8700 CPU and 32 GB RAM). The longest time was required by DOE simulations, being strictly related to the number of trials which have been tested: the final analysis with 625 experiments has taken about 1 h. All other simulations have been performed in few seconds.

## Discussion

The methodology used by the authors to establish the initial conditions has been a sort of “trial and error” where a wide spectrum of possibilities has been inquired. As such, the procedure is heavily biased by the choice of input variables to be varied with the respective range. A promising alternative approach could be based on evolutionary algorithms where the system is able to “auto-tune” itself to individuate the best solution (Dasgupta and Michalewicz, 1997; Datoussaid et al., 2002).

The first part of this work concerns the creation of an articulated multibody model suitable for the main purpose that is the analysis of a fall from a given height, knowing only the final actual position. First of all, segments, representing body parts, and connection joints between them have been created. Inertial and geometrical properties of segments were based on anthropometric data calculated from the victim height and weight, through regression equations. However, it is quite obvious that two variables are very few to fully determine body segments geometry and inertial properties; more accurate results could be obtained through a deeper examination of the victim anthropometry, for example by means of laser scanning (Pandis and Bull, 2017), CT scan or X-ray coupled to morphing methodologies (Pascoletti et al., 2020).

Connection articular joints have been modeled with classical mechanical joints or with a combination of these; some joint's degrees of freedom have been neglected since they demonstrated to undergo null or very limited movements. This simplification could not hold when analyzing other cases of fall/accidents.

The effective operation of joints has been guaranteed by the implementation of passive resistive properties retrieved from literature. Many joints have been modeled with a non-linear elastic behavior (Table 3); while for all of these a similar moment-rotation relationship was identified (Figure 2), different formulations have been chosen by the authors depending on the analyzed joint. So for some of these an exponential law has been implemented, while for the others a polynomial has been preferred. The choice between a resistive law or a linear stiffness values was based both on data available in literature and on the relative importance of joint motions with respect to the whole movement of the body.

Validation of numerical models it's a key point for their application. Human multibody model validation is not so trivial, mainly due to the problems in raising appropriate experimental data or to the possibility of performing necessary tests (Griffin, 2001; Anderson et al., 2007; Henninger et al., 2010; Lund et al., 2012).

In this work the model has been validated reproducing different fall scenarios and comparing them with results obtained by Hajiaghamemar et al. (2015) with a Hybrid III dummy. The validation process was both qualitative, for what concerns the kinematic analysis and quantitative, with reference to head impact force parameter.

For the model here presented, the performed validation should be deemed sufficient, having taken into account that all inertial and geometrical properties were obtained from well-known regression laws as well as resistive joints properties were the results of a comparison between many experimental results performed over last 40 years.

The model here introduced is not able to simulate trauma and injuries, and the corresponding energy absorption, therefore it behaves more elastically compared to the actual body response. Nonetheless, the likelihood of injuries can be established, on the basis of injury criteria (King, 2000, 2001; Prasad et al., 2010; Zanetti et al., 2014; Aldieri et al., 2018), verifying if there is a good agreement with legal medicine report.

The final objective function does not take into account appendicular skeleton movements (lower arms, lower limbs and feet): this result agrees with findings from other researcher who demonstrated the respective negligible influence (Milanowicz and Kedzior, 2017). With reference to this aspect, results of the final optimum configuration (Figure 6) have shown that the maximum errors are associated to the position of arms (the maximum distance was detected for the lower arms segments). Nevertheless, the unperfect recovery of this parts to the actual final configuration does not affect the achievement of the global orientation and position of the numerical model and so it can be neglected without loss of precision for the main objective of the study.

All the procedure has been here developed and tuned for the case of a fall from a height. Despite the application of the developed model to a single case study, is the authors' opinion that the model can be generalized to study different forensic backgrounds. Indeed, for this kind of applications, where the input parameters are final configuration's evidences, the whole procedure is the same and so the model application is quite straightforward once inertial and geometrical properties have been tuned to the person specific characteristics.

## Conclusions

This article illustrates a well-established approach where a validated multibody numerical model is used to simulate the dynamics of a human body, given its initial conditions. Special care has been paid to the accurate simulation of the passive properties of articular joints, reporting the respective elastic behavior in detail. In the specific case here analyzed, the dynamic analysis has allowed establishing the position of the victim prior to the fall and, more important, that a voluntary action had to be included in the model (in the form of an initial velocity at the central torso) in order to justify the final position of the victim. The result of the analysis was somehow unexpected since at a first glance the victim position seemed quite odd and unlikely, leaving the suspect that it had been moved after death. On the whole, a demonstration has been given of how biomechanics can give a contribution to the forensic analysis of a fall from height, together with legal medicine, suggesting that the best approach should be multidisciplinary.

## Data Availability Statement

The datasets generated for this study are available on request to the corresponding author.

## Author Contributions

GP and DC have set up the numerical model. EZ has discussed model details with GP and DC, and she has supervised the whole work with FC and PC. PC has analyzed and organized all experimental data.

### Conflict of Interest

DC was employed by the company MSC Software. The remaining authors declare that the research was conducted in the absence of any commercial or financial relationships that could be construed as a potential conflict of interest.

## Supplementary Material

Video 1 This video shows the simulation of a fall from a height of a human subject. The sequence of falling here represented is referred to the best parameters combination which best match to the actual final configuration (wireframe model in this video).
